# A multicenter, randomized, double-blind trial comparing LY01011, a biosimilar, with denosumab (Xgeva®) in patients with bone metastasis from solid tumors

**DOI:** 10.1016/j.jbo.2025.100661

**Published:** 2025-01-28

**Authors:** Mingchuan Zhao, Xichun Hu, Pengpeng Zhuang, Aiping Zeng, Yan Yu, Zhendong Chen, Hongmei Sun, Weihua Yang, Lili Sheng, Peijian Peng, Jingfen Wang, Tienan Yi, Minghong Bi, Huaqiu Shi, Mingli Ni, Xiumei Dai, Changlu Hu, Hongjie Xu, Dongqing Lv, Qingshan Li, Kaijian Lei, Xia Yuan, Ou Jiang, Xicheng Wang, Baihui Hu, Zhe Hou, Zhaoping Su, Song Zheng, Ming Zhou, Changlin Dou

**Affiliations:** a Fudan University Shanghai Cancer Center, PR China; b Shandong Boan Biotechnology Co., Ltd., PR China; c Affiliated Tumor Hospital of Guangxi Medical University, PR China; d Harbin Medical University Cancer Hospital, PR China; e the Second Affiliated Hospital of Anhui Medical University, PR China; f Jiamusi Cancer Hospital, PR China; g Shanxi Provincial Cancer Hospital, PR China; h The First Affiliated Hospital of Wannan Medical College Yijishan Hospital, PR China; i Fifth Affiliated Hospital of Sun Yat-sen University, PR China; j Linyi Cancer hospital, PR China; k Xiangyang Central Hospital, PR China; l The First Affiliated Hospital of Bengbu Medical College, Bengbu, PR China; m First Affiliated Hospital of Gannan Medical University, PR China; n Luoyang Central Hospital Affiliated to Zhengzhou University, PR China; o Xuzhou Central Hospital, PR China; p Department of Medical Oncology, Anhui Provincial Cancer Hospital, PR China; q The Affiliated Hospital of Bei-hua University, PR China; r Taizhou hospital of Zhejiang Province, PR China; s Affiliated Hospital of Chengde Medical University, PR China; t Second People’s Hospital of Yibin, PR China; u Huizhou Central People’s Hospital, PR China; v The Second People’s Hospital of Neijiang, PR China; w The First Affiliated Hospital/School of Clinical Medicine of Guangdong Pharmaceutical University, PR China; x Shandong Luye Pharmaceutical Co., Ltd., PR China

**Keywords:** Bone metastasis, Breast cancer, Denosumab, LY01011, Receptor activator of NF-κB ligand (RANKL), Urinary N-telopeptide to urinary creatinine (uNTx/uCr) ratio

## Abstract

**Introduction:**

Denosumab (Xgeva®) is a standard treatment for the prevention of skeletal-related events (SREs) in patients with bone metastases (BM). This trial was designed to assess the equivalence of LY01011 to denosumab in terms of efficacy and safety.

**Materials and methods:**

Eligible patients with BM from solid tumors were randomized at a 1:1 ratio to receive 120 mg of LY01011 or 120 mg of denosumab subcutaneously every four weeks during a 12-week double-blind treatment period, and then all enrolled patients continued to receive LY01011 until week 53. The primary endpoint was the natural logarithm of change of the urinary N-terminal crosslinked telopeptide of type I collagen level normalized to the urine creatinine level (uNTX/uCr) at week 13 from baseline. Other endpoints included the uNTX/uCr ratio, serum bone-specific alkaline phosphatase level alteration, status of anti-drug antibodies and neutralizing antibodies, adverse events and SREs.

**Results:**

850 eligible patients were randomized into the LY01011 group (n = 424) or the denosumab group (n = 426). The least-squares means (SEs) of the natural logarithms of the changes in the uNTX/uCr ratios at week 13 from baseline were −1.810 (0.0404) in the LY01011 group and −1.791 (0.0406) in the denosumab group. The LSM difference [90 % CI] between two arms was −0.019 [-0.110, 0.073] within the equivalence margins (−0.135, 0.135) and met the predetermined primary endpoint. The AEs, ADAs and the PK data showed no statistically significant difference.

**Conclusions:**

This study demonstrated the equivalent efficacy and safety of LY01011 to denosumab in patients with BM.

## Highlights

• Complications of bone metastases, skeletal-related events (SREs), can lead to functional limitations, physical deterioration and even death.
• Denosumab (Xgeva®) is used for the prevention of SREs in patients with bone metastases.
• LY01011, a denosumab biosimilar, demonstrated that it had comparable efficacy to that of the reference drug in patients with bone metastases.

## 1 Introduction

Solid tumors have become one of the main causes of human death and a major public health problem worldwide. As bone is the third most prevalent site of metastasis, after the liver and lung, the increasing incidence of solid tumors has led to an increasing incidence of bone metastases (BM).[1] BM, also known as osseous metastasis, most commonly occurs in specific cancer types, namely, prostate (85%), breast (70%), lung (40%), and kidney (40%) cancers and multiple myeloma (95%).[2] Bone metastases often lead to skeletal complications known as skeletal-related events (SREs), such as pathological fracture, necessity for radiotherapy (RT) to bone, necessity for surgery to bone, spinal cord compression and hypercalcemia. SREs usually restrict the daily activities and social functions of patients, leading to worse quality of life, increased health care expenditures and a poor prognosis [3].

Treatment of bone metastases is mainly aimed at preventing disease progression and providing symptom palliation. Several kinds of treatments may be involved, such as external-beam RT, surgical intervention, endocrine treatments, chemotherapy, targeted and immunological therapies, and bone-targeted agents (BTAs). The most common BTAs that are currently available for the treatment of BM are bisphosphonates and denosumab.[4] At the site of bone metastasis, osteoclasts are activated by receptor activator of NF-κB ligand (RANKL)[5]. Denosumab is a monoclonal antibody that binds avidly to RANKL, preventing its interaction with its receptor RANK and causing rapid suppression of bone resorption. [2] Denosumab (Xgeva®) is a standard treatment for the prevention of skeletal-related events (SREs) in patients with bone metastases.

Denosumab (Xgeva®) was first approved for the treatment of SREs in patients with bone metastases in 2010 by the United States Food and Drug Administration.[6] Subsequently, denosumab was also indicated for the treatment of adults and skeletally mature adolescents with giant cell tumors of bone, when they are unresectable or when surgical resection is likely to result in severe morbidity, and for the treatment of hypercalcemia of malignancies that are refractory to bisphosphonate therapy. Denosumab (Xgeva®) was approved in 2020 in China for the same indications [7].

LY01011 was developed by Shandong Boan Biotechnology Co., Ltd., and is a biosimilar of denosumab (Xgeva®). LY01011 was demonstrated to have similar characteristics to those of denosumab in terms of pharmacokinetics, pharmacodynamics, safety, tolerance, and immunogenicity in a previous phase I study in healthy volunteers[8]. LY06006, a denosumab biosimilar developed by Boan Biotech, was approved by the China NMPA in November 2022 as a biosimilar of Prolia® and is the first marketed denosumab biosimilar worldwide. A multicenter, randomized, double-blind, placebo-controlled phase III study (NCT05060406) of LY06006 in postmenopausal women with osteoporosis at high risk for fracture has been completed in China. Another MRCT (NCT05853354), which compares LY06006 with the EU-approved Prolia® in a similar population, has been conducted in Europe, the US and Japan since April 2023. The current study was designed to verify the equivalence of LY01011 and denosumab in patients with bone metastasis from solid tumors.

## 2 Patients and methods

### 2.1 Study population

Patients aged 18–80 years who were diagnosed with bone metastases from solid tumors were enrolled. The main inclusion criteria were as follows: patients must voluntarily sign a written informed consent form, and the patient had a solid tumor confirmed by pathological examination; at least one documented bone metastasis confirmed by computed tomography, magnetic resonance imaging or pathology (bone biopsy); an Eastern Cooperative Oncology Group performance status ≤2; and adequate organ functions at baseline. The main exclusion criteria were as follows: prior treatment with denosumab or other RANKL-targeted therapeutic drugs; previous treatment with any bone-modifying agents (including intravenous or oral bisphosphonates) for advanced tumor disease; orthopedic surgery or bone-related radiation therapy within 1 month prior to the first dose; bone radioisotope therapy within 6 months prior to the first dose; planned radiation therapy or surgery to bone during the study period; past or ongoing osteomyelitis or osteonecrosis of the jaws (ONJ); an active dental or jaw condition requiring oral surgery; nonhealed dental or oral surgery or any planned invasive dental procedure during the study period; primary central nervous system malignancy; central nervous system metastases that had failed local therapy; and asymptomatic brain metastases or clinically stable brain metastases (not requiring steroids and other therapy for ≥ 28 days).

This clinical trial followed the provisions of the Declaration of Helsinki, the International Conference on Harmonization E6 Guidelines on Good Clinical Practice, and local regulatory requirements. Written informed consent was obtained from all patients at the screening visits before the initiation of any further procedures. Every mandatory document, such as the protocol, case report form, and informed consent document, was reviewed and approved by the ethics committee of the corresponding hospital.

### 2.2 Study design and treatment

This was a multi-centered, randomized, double-blind, active-comparator, parallel-controlled phase III trial conducted in China. The study was registered at clinicaltrials.gov (NCT04859569).

**Fig. 1:**
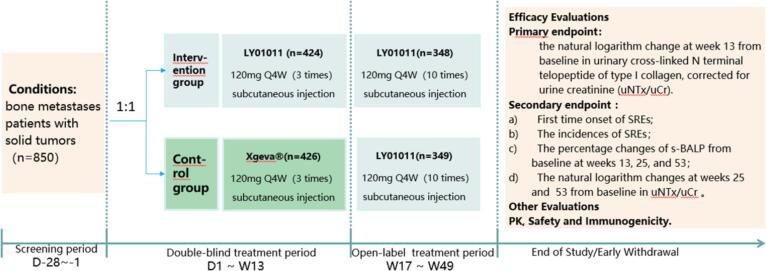
Study Design.

A total of 556–850 patients with bone metastases from solid tumors were enrolled. After screening, the eligible patients were randomized at a 1:1 ratio to receive 120 mg of LY01011 or 120 mg of denosumab subcutaneously every four weeks. Following the completion of three doses of 120 mg of LY01011 or denosumab during a 12-week double-blind treatment period (DBTP), and then all enrolled patients continued to receive LY01011 every 4 weeks up to 10 times. (Fig. 1) Twenty-eight days after the last injection, the patients completed the last regular visit to complete the safety follow-up. At least 400 IU of vitamin D and 500 mg of calcium should have been taken daily. Other treatments targeting bone metastases, such as denosumab (unplanned) and bisphosphonates, were contraindicated during the study period, while antitumor drugs and antitumor therapies not targeting bone metastases were allowed.

During the trial, blood testing and physical examinations were performed according to the trial procedures specified in the protocol for safety evaluation, and samples for pharmacokinetics, pharmacodynamics and immunogenicity testing were collected.

### 2.3 Efficacy evaluation

The efficacy parameters included in the study were the urinary N-terminal crosslinked telopeptide of type I collagen level normalized to the urine creatinine level (uNTX/uCr), the serum bone-specific alkaline phosphatase (s-BALP) level and skeletal-related events (SREs).

The primary endpoint of efficacy evaluation was the natural logarithm of change of the uNTX/uCr ratio at week 13 from baseline. The secondary endpoints of efficacy evaluation included the time to the first on-study skeletal-related event (SRE) (time frame: from baseline to week 53), the incidence of SREs (time frame: from baseline to week 53), the percent change in the s-BALP level (time frame: from baseline to weeks 13, 25, and 53), and the natural logarithm of the change in the bone turnover marker uNTX/uCr ratio (time frame: from baseline to weeks 25 and 53). The SREs were recorded and analyzed.

The time points for the collection of urine samples were as follows: predose at weeks 1, 5, 9, 13, 17, 25, and 37 and at weeks 2 and 53 (no dose administration). The time points for the collection of blood samples for s-BALP measurement were as follows: predose at weeks 1, 5, 9, 13, 25, and 37 and at weeks 2 and 53 (no dose administration).

### 2.4 Safety evaluation

Adverse events (AEs) that occurred between signing informed consent and the end of the safety follow-up period were recorded in medical records and evaluated according to the National Cancer Institute Common Terminology Criteria for Adverse Events version 5.0. All AEs were treated as instructed and monitored rigorously throughout the study. Osteonecrosis of the jaw and atypical subtrochanteric and diaphyseal femoral fractures were identified as adverse events of special interest (AESIs).

Safety profiles included data on AEs, including treatment-emergent adverse events (TEAEs), treatment-emergent serious adverse events (TESAEs), and AESIs; vital signs; physical examination results; 12-lead electrocardiogram results; and laboratory test results. AEs were coded via MedDRA Version 25.0.

### 2.5 Pharmacokinetic evaluation

Patients who consented to the pharmacokinetic (PK) evaluation were enrolled. Blood samples for PK evaluations were collected from each patient before drug administration at weeks 1, 5, 9, 13, 17 and 21. Serum concentrations of LY01011/denosumab were tested in three milliliters of blood at each time point.

### 2.6 Immunogenicity evaluation

Blood samples were collected from each patient for immunogenicity evaluation predose at weeks 1, 5, 13, and 21 and at week 53 (no dose administration). Four milliliters of blood was collected at each time point to test for anti-drug antibodies (ADAs) and neutralizing antibodies (Nabs) and to measure the titer of ADAs when ADAs were positive.

### 2.7 Sample size and statistical analysis

Assuming that the population standard deviation was approximately 0.5, based on the data from the study of denosumab (Xgeva®) in Asian solid tumor patients (NCT01920568/114273), with equivalence margins (−0.135, 0.135) to meet the requirements of the regulator [9], 80% certainty, and a ratio of 1:1 for the two groups, and taking into account a 15% dropout rate, the total number of enrolled patients was calculated to be 556 patients (test group: LY01011, 278 patients; control group: denosumab, 278 patients).

The population standard deviation was reestimated when 60% of all the enrolled patients met the predetermined primary endpoint. The sample size would not be adjusted if the population standard deviation was below 0.5; if the population standard deviation was within the range of 0.5 and 0.62, the sample size would be adjusted to correspond to 80% certainty, with the adjusted maximum sample size of no more than 850 cases. In this trial, the population standard deviation was reestimated in a blinded manner and was found to be 1.155; therefore, the sample size was increased to 850 patients.

Continuous variables are described by the number of instances, mean, standard deviation, median, Q1, Q3, minimum and maximum. For log-transformed indicators, the number of cases, arithmetic mean, standard deviation, coefficient of variation, median, Q1, Q3, minimum, maximum, geometric mean and geometric coefficient of variation were calculated. Categorical variables are described by the number and percentage of each category.

Primary efficacy evaluation: After filling in the missing values of the uNTx/uCr ratio at 13 weeks using the LOCF (last observation carried forward) method, the natural logarithms of the relative changes in the uNTx/uCr values at 13 weeks from baseline were analyzed using analysis of covariance (ANCOVA). In the model, the relative change in the 13-week uNTx/uCr ratio from baseline after taking the natural logarithm was the dependent variable, the group and random stratification factors (tumor type) were the fixed effects, and the natural logarithm of the baseline uNTx/uCr ratio was the covariate. The least-squares mean (LSM) and its 90% CI were calculated for each group according to the model, and the difference between the LSMs in the LY01011 and denosumab groups and its 90% CI were also calculated. Equivalence was fulfilled if the 90% CI of the difference fell exactly within the equivalence margins (−0.135, 0.135). Subgroup analyses of the primary endpoints were performed based on age, sex, and tumor type.

Statistical analyses were performed by using SAS version 9.4. All statistical tests were two-sided, and a p value of <0.05 was considered to indicate statistical significance for the differences tested (unless otherwise specified).

## 3 Results

### 3.1 Demographic and baseline characteristics

**Fig. 2:**
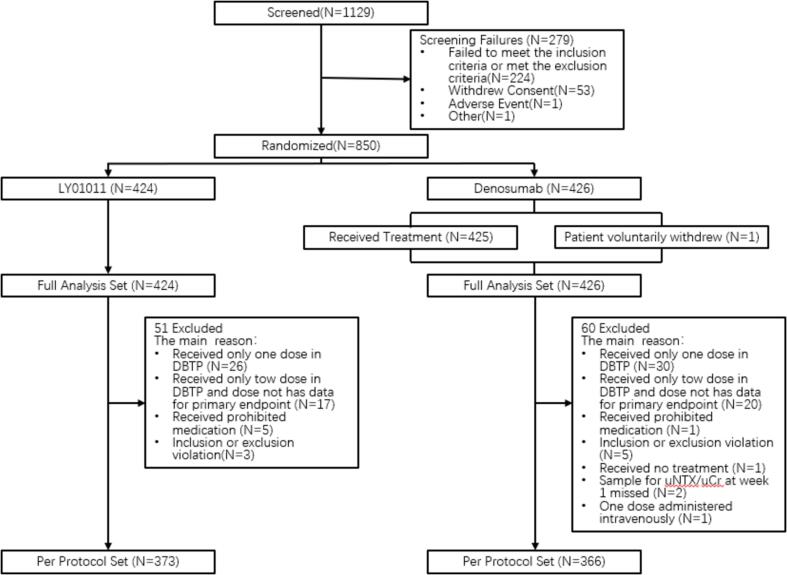
Patient Disposition.

Following the screening, 850 eligible patients were enrolled and randomized at a 1:1 ratio to each group, with 424 patients in the LY01011 group and 426 patients in the denosumab group. All randomized patients were included in the full analysis set (FAS). The per protocol set (PPS) included 739 patients (373 and 366 patients in the LY01011 and denosumab groups, respectively (Fig. 2). The demographic and characteristics are reported based on the full analysis set (FAS) population, and the two groups were well balanced (Table 1). In the FAS population, the mean age (SD) was 57.2 (10.77) years, 361 patients (42.5%) were males, and 461 patients (54.2%) had lung cancer as the primary tumor. The baseline uNTx/uCr ratios and s-BALP levels were comparable between the 2 treatment groups.

**Table 1:** Demographic and Baseline Characteristics.

	LY01011 (n = 424)	Denosumab (n = 426)	Total (n = 850)
Age, Mean (SD), y	57.7 (10.38)	56.8 (11.14)	57.2 (10.77)
Sex, No. (%)			
Male	170 (40.1)	191 (44.8)	361 (42.5)
Female	254 (59.9)	235 (55.2)	489 (57.5)
Ethnicity, No. (%)			
Han Chinese	406 (95.8)	406 (95.3)	812 (95.5)
Height, Mean (SD), cm	161.76 (7.990)	162.88 (8.188)	162.32 (8.104)
Weight, Mean (SD), kg	60.47 (10.104)	62.40 (10.980)	61.44 (10.591)
BMI, Mean (SD), kg/m^2^	23.06 (3.115)	23.45 (3.327)	23.26 (3.228)
Location of primary tumor, No. (%)			
Breast	119 (28.1)	120 (28.2)	239 (28.1)
Lung	230 (54.2)	231 (54.2)	461 (54.2)
Other	75 (17.7)	75 (17.6)	150 (17.6)
ECOG performance status, No. (%)			
0	81 (19.1)	93 (21.9)	174 (20.5)
1	319 (75.2)	319 (75.1)	638 (75.1)
2	24 (5.7)	13 (3.1)	37 (4.4)
Data missing	0	1 (0.2)	1 (0.1)
uNTx/uCr, mean (SD), nM BCE/mM creatinine	66.504 (70.2052)	66.502 (77.0611)	66.503 (73.6693)
s-BALP, mean (SD), μg/L	25.015 (27.2557)	27.091 (40.7592)	26.053 (34.6663)

Abbreviations: BMI, body mass index (calculated as weight in kilograms divided by height in meters squared); ECOG, Eastern Cooperative Oncology Group; uNTX/uCr, natural logarithm-transformed urinary N-telopeptide/creatinine ratio; s-BALP, natural logarithm-transformed serum bone-specific alkaline phosphatase concentration; SREs, skeletal-related events.

### 3.2 Efficacy

**Fig. 3:**
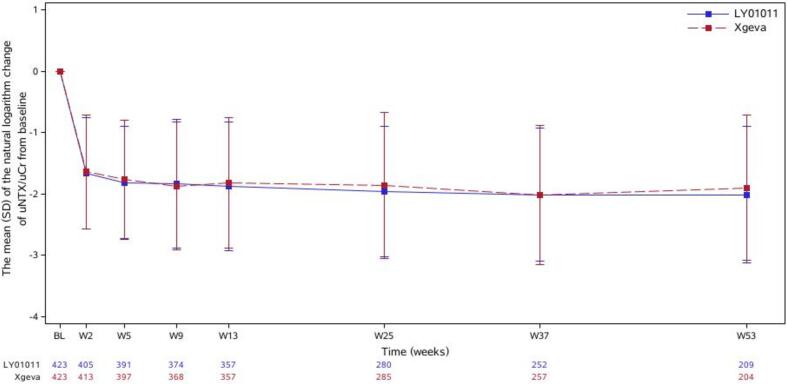
Time courses of the mean (SD) of the natural logarithm changes of uNTX/uCr from baseline (entire study; FAS).

Significant decreases in the uNTX/uCr ratios could be observed in the two groups at 1 week after administration, with a maximum decrease in the median of up to approximately 80% from baseline, and this decreasing trend persisted until the last assessment. The trend for the natural logarithms of the changes in the uNTX/uCr ratios from baseline was similar. In the FAS (n=850), the least-squares means (LSMs) (standard errors [SEs]) of the natural logarithms of the changes in the uNTX/uCr ratios at week 13 from baseline were −1.810 (0.0404) in the LY01011 group and −1.791 (0.0406) in the denosumab group. The LSM difference [90% CI] was −0.019 [-0.110, 0.073]. This result was within the equivalence margins (−0.135, 0.135) and met the predetermined primary endpoint. In the per protocol set (PPS) (n=739), the LSM difference [90% CI] was −0.013 (−0.104, 0.079), which also met the predefined equivalence margins. Subgroup analyses showed that there were no statistically significant differences in the natural logarithms of the changes in the uNTX/uCr ratios at week 13 from baseline between the two groups in terms of sex, age (≤60 years and >60 years), or tumor type (breast cancer, lung cancer, or other) (Table 2 and Fig. 3).

**Table 2:** Primary Endpoint and Subgroup Analysis.

	Least-squares mean of the change at week 13 from baseline (SE)		Difference	
Variable	LY01011	Denosumab	(90 % CI)	*P value*
FAS	−1.810 (0.0404)	−1.791 (0.0406)	−0.019(−0.110, 0.073)	0.7380
PPS	−1.802 (0.0405)	−1.789 (0.0408)	−0.013(−0.104, 0.079)	0.8206
Sex subgroups				
Male	−1.746 (0.0684)	−1.783 (0.0645)	0.037(−0.112, 0.186)	0.6827
Female	−1.862 (0.0515)	−1.820 (0.0544)	−0.042 (−0.157, 0.073)	0.5446
Age subgroups				
Age <=60Y	−1.796 (0.0504)	−1.815 (0.0517)	0.020 (−0.096, 0.135)	0.7791
Age ＞60Y	−1.854 (0.0681)	−1.768 (0.0660)	−0.086 (−0.236, 0.064)	0.3434
Cancer subgroups				
Breast cancer	−1.667 (0.0722)	−1.741 (0.0736)	0.076 (−0.097, 0.248)	0.4706
Lung cancer	−1.999 (0.0505)	−1.979 (0.0496)	−0.020 (−0.137, 0.097)	0.7758
Other cancer	−1.702 (0.1126)	−1.523 (0.1157)	−0.179 (−0.447, 0.090)	0.2714

Abbreviations: SE, standard error; FAS, full analysis set; PPS, per protocol set.

**Fig. 4:**
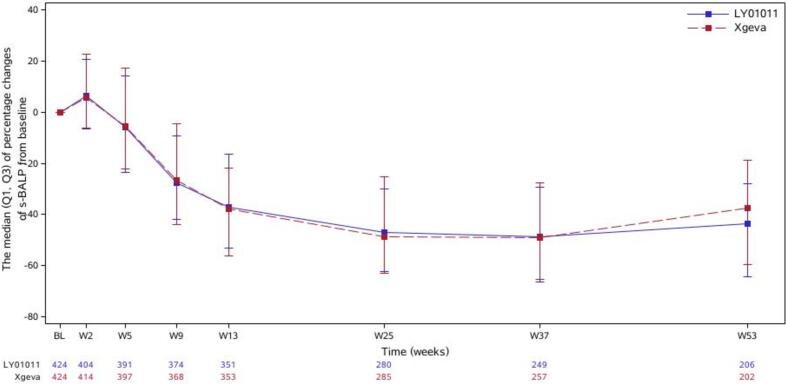
Time courses of the median (Q1, Q3) of percentage changes of s-BALP from baseline (entire study; FAS).

The s-BALP levels in both the LY01011 and denosumab groups decreased continuously from 4 weeks after the first drug administration, and the decreasing trends persisted until the last assessment. The median percent changes in the s-BALP levels at weeks 13, 25, and 53 from baseline in the two groups (LY01011 vs. denosumab) were −36.980% vs. −37.674%, −46.853% vs. −48.681%, and −43.551% vs. −37.598%, respectively (Fig. 4).

**Fig. 5:**
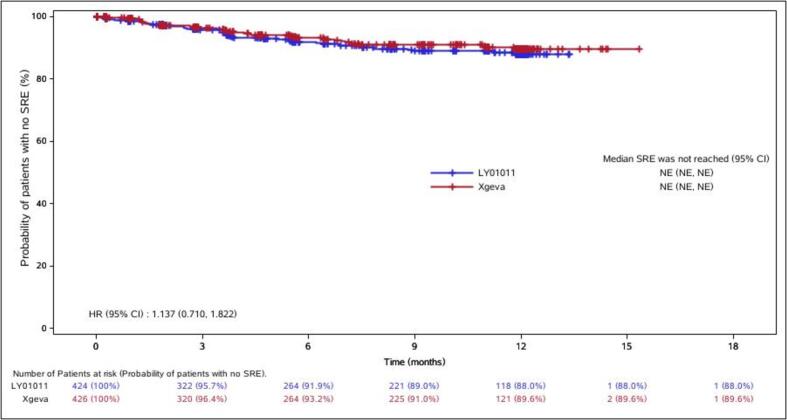
Time courses of probabilities of patients with no SRE (%) (entire study; FAS).

The incidences of SREs were also analyzed. During the DBTP, the percentages of SREs that occurred in the LY01011 and denosumab groups were 3.5% and 2.8%, respectively, and during the entire study, they were 9.0% and 7.7%, respectively. There are less SREs in the denosumab group but that the stats are not significantly different. The probabilities of the patients in the treatment groups having no SRE over time are shown in (Fig. 5).

### 3.3 Safety

Patients who received at least 1 dose of the study drugs were included in the safety analyses (424 patients in the LY01011 group and 425 patients in the denosumab group). AEs were recorded and analyzed both during the DBTP and throughout the entire study.

During the DBTP, 775 (91.3%) patients had at least one TEAE, with 389 (91.7%) and 386 (90.8%) patients having at least one TEAE in the LY01011 and denosumab groups, respectively (Table 2). The most common TEAEs (experienced by ≥ 20% of the patients in either group) were a decreased white blood cell count (32.5% vs. 34.8%), a decreased neutrophil count (32.1% vs. 33.6%), anemia (30.7% vs. 28.7%), and hypocalcemia (20.3% vs. 18.4%). The proportions of patients with TEAEs ≥ grade 3 (38.4% and 38.4%) and TESAEs (13.2% and 14.1%) were similar between the groups. A decreased neutrophil count (14.4% vs. 16.0%), a decreased white blood cell count (8.3% vs. 10.6%), and anemia (6.6% vs. 6.6%) were the most common TEAEs ≥ grade 3. The treatment-related adverse events (TRAEs) experienced by ≥ 5% of the patients in either group (LY01011 vs. denosumab) were hypocalcemia (20.0% vs. 18.1%) and hypophosphatemia (12.0% vs. 13.6%) (Table 3).

**Table 3:** Safety analysis of adverse events during the DBTP (safety set).

	LY01011 (n = 424)	Denosumab (n = 425)	Total (n = 849)
All TEAEs	389 (91.7 %)	386 (90.8 %)	775 (91.3 %)
Treatment-related	174 (41.0 %)	179 (42.1 %)	353 (41.6 %)
TEAEs ≥ Grade 3	163 (38.4 %)	163 (38.4 %)	326 (38.4 %)
Treatment-related	23 (5.4 %)	19 (4.5 %)	42 (4.9 %)
TEAEs leading to study drug suspension	12 (2.8 %)	8 (1.9 %)	20 (2.4 %)
Treatment-related	2 (0.5 %)	0	2 (0.2 %)
TEAEs leading to study drug discontinuation	10 (2.4 %)	20 (4.7 %)	30 (3.5 %)
Treatment-related	0	4 (0.9 %)	4 (0.5 %)
TESAEs	56 (13.2 %)	60 (14.1 %)	116 (13.7 %)
Treatment-related	3 (0.7 %)	2 (0.5 %)	5 (0.6 %)
TEAEs leading to death	15 (3.5 %)	13 (3.1 %)	28 (3.3 %)
Treatment-related	0	0	0
AESIs	0	0	0
TEAEs in ≥ 20 % of the patients in either group			
White blood cell count decreased	138 (32.5 %)	148 (34.8 %)	286 (33.7 %)
Neutrophil count decreased	136 (32.1 %)	143 (33.6 %)	279 (32.9 %)
Anemia	130 (30.7 %)	122 (28.7 %)	252 (29.7 %)
Hypocalcemia	86 (20.3 %)	78 (18.4 %)	164 (19.3 %)
Treatment-related TEAEs in ≥ 5 % of the patients in either group			
Hypocalcemia	85 (20.0 %)	77 (18.1 %)	162 (19.1 %)
Hypophosphatemia	51 (12.0 %)	58 (13.6 %)	109 (12.8 %)
TEAEs ≥ Grade 3 in ≥ 5 % of the patients in either group			
Neutrophil count decreased	61 (14.4 %)	68 (16.0 %)	129 (15.2 %)
White blood cell count decreased	35 (8.3 %)	45 (10.6 %)	80 (9.4 %)
Anemia	28 (6.6 %)	28 (6.6 %)	56 (6.6 %)
TESAEs in ≥ 1 % of the patients in either group			
White blood cell count decreased	9 (2.1 %)	7 (1.6 %)	16 (1.9 %)
Malignant neoplasm progression	7 (1.7 %)	7 (1.6 %)	14 (1.6 %)
Platelet count decreased	5 (1.2 %)	7 (1.6 %)	12 (1.4 %)
Anemia	3 (0.7 %)	8 (1.9 %)	11 (1.3 %)
Neutrophil count decreased	6 (1.4 %)	4 (0.9 %)	10 (1.2 %)
Treatment-related TESAEs			
Diarrhea	1 (0.2 %)	1 (0.2 %)	2 (0.2 %)
Vomiting	1 (0.2 %)	0	1 (0.1 %)
Nausea	1 (0.2 %)	0	1 (0.1 %)
Decreased appetite	1 (0.2 %)	0	1 (0.1 %)
Hypocalcemia	0	1 (0.2 %)	1 (0.1 %)

Abbreviations: TEAEs, treatment-emergent adverse events; TESAEs, treatment-emergent serious adverse events; AESIs, adverse events of special interest.

During the entire study, the proportions of patients who reported at least 1 TEAE were similar between the 2 groups (413 patients (97.4%) in the LY01011 group and 411 patients (96.7%) in the denosumab group). The outcomes of the TEAEs were mostly improved, stabilized, or restored to baseline levels. The most common TEAEs (experienced by ≥ 20% of the patients in either group, LY01011 vs. denosumab) included a decreased white blood cell count (46.7% vs. 47.3%), anemia (46.5% vs. 43.3%), a decreased neutrophil count (43.9% vs. 44.0%), a decreased platelet count (26.9% vs. 31.5%), hypocalcemia (30.7% vs. 27.1%), an increased aspartate aminotransferase level (26.2% vs. 22.4%), an increased alanine aminotransferase level (23.1% vs. 21.6%), hypophosphatemia (19.3% vs. 22.1%), nausea (21.9% vs. 18.6%), decreased appetite (21.9% vs. 18.4%), and asthenia (22.2% vs. 14.6%). The proportions of patients with TEAEs ≥ grade 3 (54.7% vs. 57.9%) and TESAEs (28.1% vs. 30.4%) were similar between the groups. A decreased neutrophil count (20.0% vs. 23.5%), a decreased white blood cell count (13.9% vs. 16.0%), and anemia (10.1% vs. 10.6%) were the most common TEAEs ≥ grade 3. The treatment-related TEAEs experienced by ≥ 5% of the patients in either group (LY01011 vs. denosumab) were hypocalcemia (28.1% vs. 25.9%) and hypophosphatemia (18.4% vs. 21.2%). No ONJ or any other AESIs were recognized during the entire study (Table 4).

**Table 4:** Safety analysis of adverse events during the entire study (safety set).

	LY01011 (n = 424)	Denosumab (n = 425)	Total (n = 849)
All TEAEs	413 (97.4 %)	411 (96.7 %)	824 (97.1 %)
Treatment-related	237 (55.9 %)	242 (56.9 %)	479 (56.4 %)
TEAEs ≥ Grade 3	232 (54.7 %)	246 (57.9 %)	478 (56.3 %)
Treatment-related	28 (6.6 %)	28 (6.6 %)	56 (6.6 %)
TEAE leading to study drug suspension	29 (6.8 %)	26 (6.1 %)	55 (6.5 %)
Treatment-related	4 (0.9 %)	1 (0.2 %)	5 (0.6 %)
TEAE leading to study drug discontinuation	23 (5.4 %)	37 (8.7 %)	60 (7.1 %)
Treatment-related	0	7 (1.6 %)	7 (0.8 %)
TESAEs	119 (28.1 %)	129 (30.4 %)	248 (29.2 %)
Treatment-related	3 (0.7 %)	7 (1.6 %)	10 (1.2 %)
TEAEs leading to death	53 (12.5 %)	48 (11.3 %)	101 (11.9 %)
Treatment-related	0	0	0
AESIs	0	0	0
TEAEs in ≥ 20 % of the patients in either group			
White blood cell count decreased	198 (46.7 %)	201 (47.3 %)	399 (47.0 %)
Anemia	197 (46.5 %)	184 (43.3 %)	381 (44.9 %)
Neutrophil count decreased	186 (43.9 %)	187 (44.0 %)	373 (43.9 %)
Platelet count decreased	114 (26.9 %)	134 (31.5 %)	248 (29.2 %)
Hypocalcemia	130 (30.7 %)	115 (27.1 %)	245 (28.9 %)
Aspartate aminotransferase level increased	111 (26.2 %)	95 (22.4 %)	206 (24.3 %)
Alanine aminotransferase level increased	98 (23.1 %)	92 (21.6 %)	190 (22.4 %)
Hypophosphatemia	82 (19.3 %)	94 (22.1 %)	176 (20.7 %)
Nausea	93 (21.9 %)	79 (18.6 %)	172 (20.3 %)
Decreased appetite	93 (21.9 %)	78 (18.4 %)	171 (20.1 %)
Asthenia	94 (22.2 %)	62 (14.6 %)	156 (18.4 %)
Treatment-related TEAEs in ≥ 5 % of the patients in either group			
Hypocalcemia	119 (28.1 %)	110 (25.9 %)	229 (27.0 %)
Hypophosphatemia	78 (18.4 %)	90 (21.2 %)	168 (19.8 %)
TEAEs ≥ Grade 3 in ≥ 5 % of the patients in either group			
Neutrophil count decreased	85 (20.0 %)	100 (23.5 %)	185 (21.8 %)
White blood cell count decreased	59 (13.9 %)	68 (16.0 %)	127 (15.0 %)
Anemia	43 (10.1 %)	45 (10.6 %)	88 (10.4 %)
Platelet count decreased	30 (7.1 %)	40 (9.4 %)	70 (8.2 %)
Malignant neoplasm progression	39 (9.2 %)	28 (6.6 %)	67 (7.9 %)
Lymphocyte count decreased	6 (1.4 %)	22 (5.2 %)	28 (3.3 %)
TESAEs in ≥ 1 % of the patients in either group			
Malignant neoplasm progression	39 (9.2 %)	29 (6.8 %)	68 (8.0 %)
White blood cell count decreased	14 (3.3 %)	14 (3.3 %)	28 (3.3 %)
Platelet count decreased	11 (2.6 %)	16 (3.8 %)	27 (3.2 %)
Pneumonia	10 (2.4 %)	13 (3.1 %)	23 (2.7 %)
Neutrophil count decreased	10 (2.4 %)	11 (2.6 %)	21 (2.5 %)
Anemia	6 (1.4 %)	9 (2.1 %)	15 (1.8 %)
Cerebral infarction	5 (1.2 %)	5 (1.2 %)	10 (1.2 %)
Respiratory failure	4 (0.9 %)	5 (1.2 %)	9 (1.1 %)
Metastases to meninges	0	5 (1.2 %)	5 (0.6 %)
Treatment-related TESAEs			
Hypocalcemia	0	2 (0.5 %)	2 (0.2 %)
Hypokalemia	0	2 (0.5 %)	2 (0.2 %)
Nausea	1 (0.2 %)	1 (0.2 %)	2 (0.2 %)
Diarrhea	1 (0.2 %)	1 (0.2 %)	2 (0.2 %)
Vomiting	1 (0.2 %)	0	1 (0.1 %)
Toothache	0	1 (0.2 %)	1 (0.1 %)
Decreased appetite	1 (0.2 %)	0	1 (0.1 %)
Pneumonia	0	1 (0.2 %)	1 (0.1 %)

Abbreviations: TEAEs, treatment-emergent adverse events; TESAEs, treatment-emergent serious adverse events; AESIs, adverse events of special interest.

### 3.4 Pharmacokinetic evaluations

In the pharmacokinetic concentration set, the predose plasma concentrations increased at weeks 5, 9, and 13 in both drug groups; the rate of increase decreased from week 16 to week 17, and a steady state was reached at week 21.

**Fig. 6:**
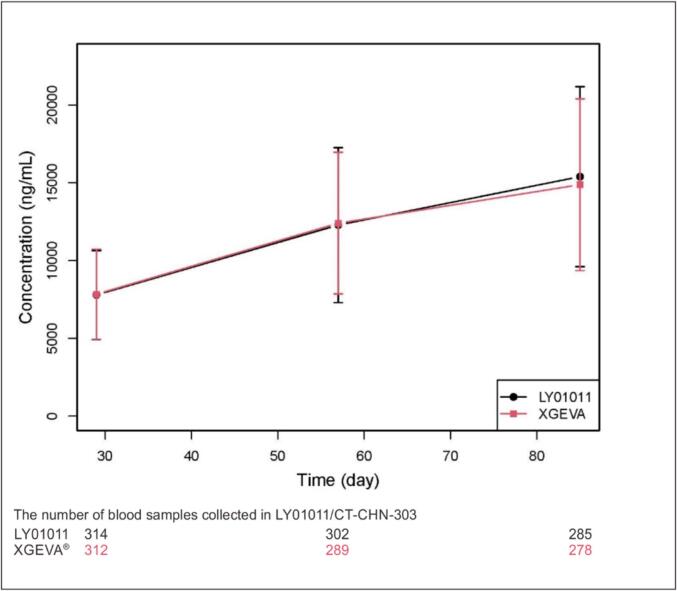
Mean blood concentration–time profiles of the study drugs.

The blood concentrations of the study drugs before administration were similar in the two groups at weeks 5, 9 and 13. The mean blood concentration–time profiles are shown in (Fig. 6).

According to the DBTP data, LY01011 and denosumab PK data was described by a two-compartment model with first order absorption and parallel linear and nonlinear elimination, and the results showed that there were no significant differences in the main PK parameters of clearance (P = 0.6879) or central volume of distribution (P = 0.9984) between LY01011 group and denosumab group.

### 3.5 Immunogenicity evaluations

In the present study, all the patients tested ADA negative at baseline prior to the study drug administration. In the LY01011 group, one patient was reported to be ADA positive and to have an ADA antibody titer of 80 at week 5, but his blood tests for ADAs were negative at weeks 13, 21 and 53. In the denosumab group, one patient was also reported to be ADA positive at week 21, with an ADA antibody titer <5. No patients were Nab positive.

## 4 Discussion

This multicenter, randomized, double-blind trial was designed to verify the equivalence of LY01011 with denosumab in terms of its efficacy and safety in patients with bone metastasis from solid tumors.

This trial met its primary endpoint by demonstrating that LY01011 had an equivalent effect on reducing the uNTX/uCr ratio at week 13 compared with denosumab, which is considered to indicate an equivalent effect on the prevention of SREs in patients with bone metastases. The reasons for this are as follows.

N-terminal crosslinked telopeptide of type I collagen (NTX) is one of the products generated by the breakdown of type 1 collagen by acid phosphatases [10]. The alpha-2 isoform of NTX is bone derived and is usually used as a biomarker of bone resorption [11]. NTX in the blood is not metabolized by the liver but is excreted directly into the urine via the kidneys in its original form [12]. The urinary level of N-terminal crosslinked telopeptide of type I collagen normalized to the urine creatinine level (uNTX/uCr) has been demonstrated to correlate with the occurrence of bone-related events in several clinical studies of bone metastases. [13], [14], [15], [16] Changes in the levels of NTX as a PD biomarker are accepted by different health authorities, e.g., the US FDA and China NMPA. As NTX levels are highly variable in the target population, in this study, changes in the uNTX/uCr ratio from baseline were statistically analyzed as the natural logarithm and percent. [9], [17] The natural logarithm of the change in the uNTX/uCr ratio from baseline decreased after the first dose and stabilized at week 13 after multiple doses.

The trends of the natural logarithms of the changes in the uNTX/uCr ratios from baseline in the two groups were similar, which confirmed the similarity between the two drugs.

Bone alkaline phosphatase (BALP) is a particular isoform of alkaline phosphatase that is found only in osteoblasts. In the context of bone metastasis, elevated bone resorption leads to the coupling of bone resorption and formation and to increased BALP levels [9]. As shown in Fig. 3, the uNTX/uCr ratio started to decrease at week 2, while Fig. 4 shows that the level of s-BALP did not decrease at week 2, which also showed that at the site of bone metastasis, osteogenesis occurred secondary to bone destruction. The trends of the percentage changes in BALP levels from baseline in the two groups were similar.

Denosumab was demonstrated to be better than zoledronic in preventing SREs in 3 pivotal phase III studies in which delays until the first and subsequent on-study SREs were chosen as the primary endpoints. In this study, during the DBTP, the percentages of SREs that occurred in the LY01011 and denosumab groups were 3.5% and 2.8%, respectively, and during the entire study, they were 9.0% and 7.7%, respectively. There were no statistically significant differences.

Before this phase III study, a randomized trial comparing LY01011, a biosimilar candidate, with the reference product denosumab in healthy Chinese individuals was conducted and showed that the PK and PD of LY01011 were similar to those of denosumab, with comparable safety and immunogenicity profiles [18]. In this phase III study, the similarities between the PK and PD of LY01011 and denosumab were demonstrated in patients with bone metastases.

The results of this phase III study showed that both the primary and secondary efficacy endpoints during the DBTP met the predefined parameters, which demonstrated that LY01011 had an equivalent efficacy to denosumab. After the DBTP, there was no significant difference between the two cohorts, which demonstrated that the efficacies of the study drugs remained stable.

TEAEs, treatment-related adverse events (TRAEs) and TESAEs were common side effects of chemotherapy both during the DBTP and the entire study, and the incidence rates were comparable between the groups (Table 2), with no unexpected adverse reactions reported. During the DBTP, the main TRAEs in both groups (LY01011 vs. denosumab) were hypocalcemia (28.1% vs. 25.9%) and hypophosphatemia (18.4% vs. 21.2%). The incidence of ONJ in this study was also lower than that on the label because the safety observation period was not as long as those of the 3 pivotal phase III studies of denosumab. The safety results for the DBTP demonstrated that LY01011 had a similar safety profile to denosumab, and the safety evaluation for the entire study showed no unexpected safety risks during drug switching after week 13 to the end of the study.

All patients tested ADA negative at baseline. Subsequently, two patients were reported to be ADA positive. No patients were Nab positive. According to the label, using an electrochemiluminescent bridging immunoassay, less than 1% (7/2758) of patients with osseous metastases who were treated with denosumab (Xgeva®) at doses ranging from 30 to 180 mg every 4 weeks or every 12 weeks for up to 3 years tested positive for binding antibodies against denosumab. The immunogenicity observed in the present study was low and similar to that of the reference product denosumab (Xgeva®), and the low risk of immunogenicity was consistent with the results of a previous study of LY01011 [8].

## 5 Conclusion

This study demonstrated that the efficacy of LY01011 in reducing the bone metabolism biomarker NTX was equivalent to that of the reference product denosumab (Xgeva®), thus meeting the primary endpoint. The safety characteristics of LY01011 and denosumab (Xgeva®) were similar, and no unexpected adverse reactions were reported. Switching therapeutic drugs after week 13 did not affect the efficacy, safety or immunogenicity profiles of either group.

Limitations

A long-term follow up study was not conducted in patients with bone metastasis from solid tumors. Considering its mechanism of action and substantial evidence from prior clinical studies, denosumab was unlikely to prolong overall survival. Therefore, the long-term observations of SREs and overall survival might not be necessary for a comparable biosimilar study. Non-Chinese participants were not included in the study which provided no information on the safety and efficacy data of LY01011 in foreign patients with bone metastasis.

## CRediT authorship contribution statement

**Mingchuan Zhao:** Writing – review & editing, Writing – original draft, Methodology, Formal analysis, Data curation, Conceptualization. **Xichun Hu:** Writing – review & editing, Resources, Project administration, Investigation, Conceptualization. **Pengpeng Zhuang:** Writing – review & editing, Writing – original draft, Project administration, Data curation, Conceptualization. **Aiping Zeng:** Writing – review & editing, Resources. **Yan Yu:** Writing – review & editing, Resources. **Zhendong Chen:** Writing – review & editing, Resources. **Hongmei Sun:** Writing – review & editing, Resources. **Weihua Yang:** Writing – review & editing, Resources. **Lili Sheng:** Writing – review & editing, Resources. **Peijian Peng:** Writing – review & editing, Resources. **Jingfen Wang:** Writing – review & editing, Resources. **Tienan Yi:** Writing – review & editing, Resources. **Minghong Bi:** Writing – review & editing, Resources. **Huaqiu Shi:** Writing – review & editing, Resources. **Mingli Ni:** Writing – review & editing, Resources. **Xiumei Dai:** Writing – review & editing, Resources. **Chang-Lu Hu:** Writing – review & editing, Resources. **Hongjie Xu:** Writing – review & editing, Resources. **Dongqing Lv:** Writing – review & editing, Resources. **Qingshan Li:** Writing – review & editing, Resources. **Kaijian Lei:** Writing – review & editing, Resources. **Xia Yuan:** Writing – review & editing, Resources. **Ou Jiang:** Writing – review & editing, Resources. **Xi-Cheng Wang:** Writing – review & editing, Resources. **Baihui Hu:** Data curation, Investigation, Visualization, Writing – review & editing. **Zhe Hou:** Conceptualization, Data curation, Investigation, Software, Writing – review & editing. **Zhaoping Su:** Data curation, Investigation, Project administration, Software, Writing – review & editing. **Song Zheng:** Data curation, Writing – review & editing. **Ming Zhou:** Data curation, Formal analysis, Funding acquisition, Methodology, Project administration, Resources, Supervision, Writing – review & editing. **Changlin Dou:** Data curation, Formal analysis, Funding acquisition, Investigation, Project administration, Resources, Supervision, Writing – review & editing.

## Declaration of competing interest

The authors declare that they have no known competing financial interests or personal relationships that could have appeared to influence the work reported in this paper.
